# Successful management of clip embolization into coronary sinus after transcatheter edge-to-edge mitral valve repair

**DOI:** 10.1093/ehjcr/ytae302

**Published:** 2024-06-27

**Authors:** Da Zhu, Yu Yang, Shouzheng Wang, XiangBin Pan

**Affiliations:** Department of Structure Heart Center, Fuwai Yunnan Hospital, Chinese Academy of Medical Sciences, Affiliated Cardiovascular Hospital of Kunming Medical University, 528 Shahebei Road, Wuhua District, Kunming 650102, China; Department of Structure Heart Center, Fuwai Yunnan Hospital, Chinese Academy of Medical Sciences, Affiliated Cardiovascular Hospital of Kunming Medical University, 528 Shahebei Road, Wuhua District, Kunming 650102, China; Department of Structure Heart Center, Fuwai Yunnan Hospital, Chinese Academy of Medical Sciences, Affiliated Cardiovascular Hospital of Kunming Medical University, 528 Shahebei Road, Wuhua District, Kunming 650102, China; Department of Structure Heart Center, Fuwai Hospital, National Center for Cardiovascular Diseases, Chinese Academy of Medical Sciences and Peking Union Medical College, 167 Beilishi Road, Xicheng District, Beijing 100037, China; Department of Structure Heart Center, Fuwai Yunnan Hospital, Chinese Academy of Medical Sciences, Affiliated Cardiovascular Hospital of Kunming Medical University, 528 Shahebei Road, Wuhua District, Kunming 650102, China; Department of Structure Heart Center, Fuwai Hospital, National Center for Cardiovascular Diseases, Chinese Academy of Medical Sciences and Peking Union Medical College, 167 Beilishi Road, Xicheng District, Beijing 100037, China

A 64-year-old man presented to hospital due to advanced heart failure, dilated cardiomyopathy, severe functional mitral regurgitation (MR) despite optimal medical treatment with NYHA IV. Transoesophageal echocardiogram (TEE) revealed a wide jet from entire A2/P2 region affecting 1–3 regions. A large indentation was noted between P1 and P2 regions (*Figure 1A* and *B*). After Heart Team discussion, patient received transcatheter edge-to-edge repair (TEER) using Valveclip system (Newmed Medical, Shanghai, China)—a Chinese-made self-locking ‘Pascal-like’ clip device (*Figure 1C*). Under the guidance of TEE, three clips were implanted. Transoesophageal echocardiogram confirmed significant MR reduction from 4+ to 1+ and with mean gradient 1 mmHg (*Figure 1C* and *D* and Supplementary material online, *Video S1*).

**Figure 1:**
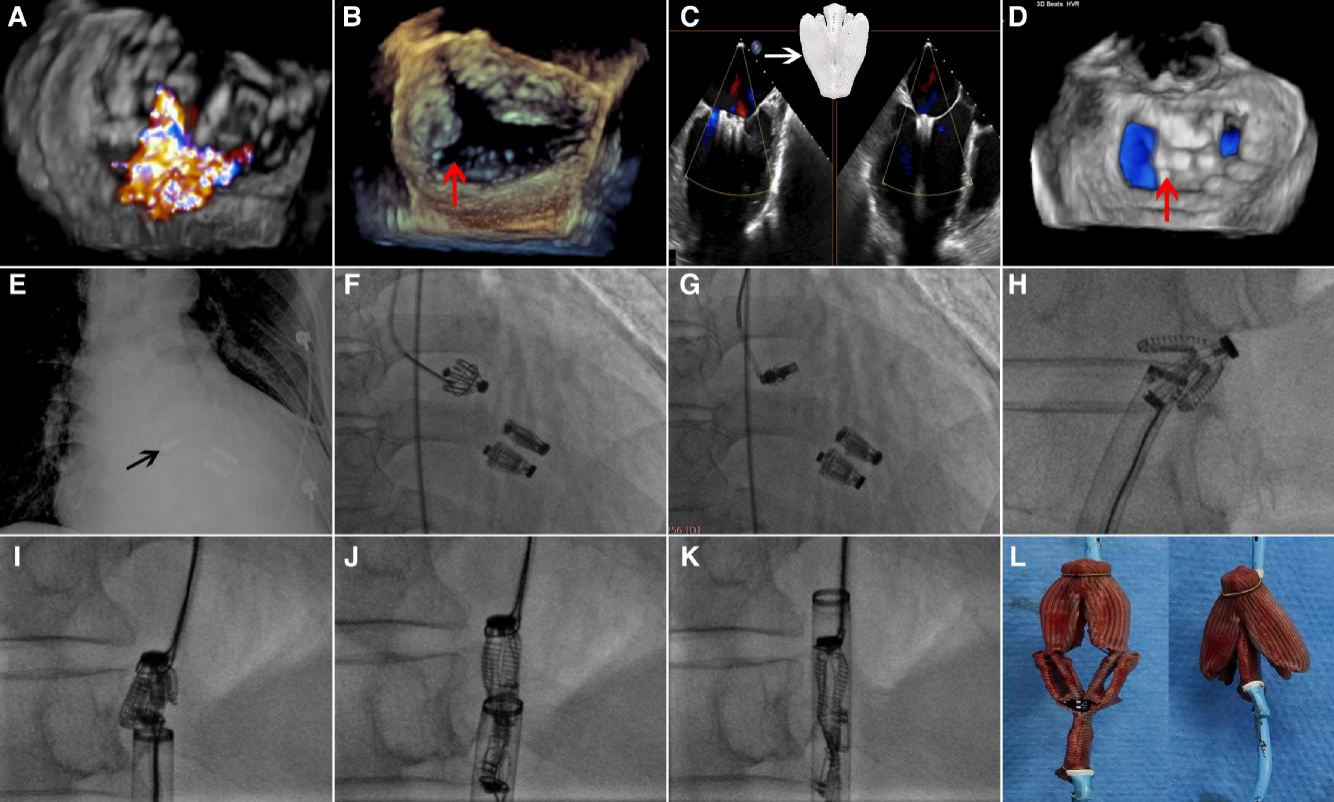
Procedural steps of percutaneous retrieval of embolized ‘Pascal-like’ clip device. (*A* and *B*) 4+ FMR was noted with big indentation at P1–2 area (red arrow); (*C* and *D*) Three Clip (white arrow) was implanted with one clip locating at indentation area (red arrow). (*E*) Bedside X-ray confirmed clip embolization (black arrow). (*F-H*) The embolized clip was pulled back into the descending aorta by gooseneck snare. (*I-K*) Using two gooseneck snares technique, clip was successfully removed through 20 F sheath. (*L*) *In vitro* demonstration of the removed ‘Pascal-like’ clip.

After being transferred to ward on the second day, the patient had a sudden onset of chest pain with orthopnoea and bradycardia. Electrocardiogram showed ST-segment elevation in the inferior lead. Bedside echocardiogram and chest X-ray (*Figure 1E*) showed that one clip had embolized into the right aortic sinus and blocked the right coronary ostium. Emergency intervention was done, and fluoroscopy confirmed the diagnosis. Through right femoral access, a 6 Fr multipurpose angiographic (MPA) catheter was forwarded to the right aortic sinus, the embolized clip was pulled back into the descending aorta by gooseneck snare (*Figure 1F–H*). The coronary angiography showed that the right coronary artery was unobstructed without coronary injury. Patient developed reperfusion arrhythmia, and the condition gradually stabilized after treatment. Because of relatively larger size in closing status of ‘Pascal-like’ clip in compared with Mitraclip (*Figure 1H*), in order to remove the clip from the aorta, the left femoral artery was punctured and 20F Gore® vascular sheath was implanted, the left radial artery access was also used through 5 F sheath. Through two MPA catheters, two gooseneck snares were used to capture the tip and the button of the clip from opposite side. By pulling two MPA catheters and snares, the clip was been manipulated into elongation status (delivery status) and then successfully retrieved back into 20 F catheter (*Figure 1I–L* and Supplementary material online, *Video S2*). Transoesophageal echocardiogram revealed 2+ residual mitral regurgitation. At the 1-month follow-up, patient’s symptoms were improved, and no other procedure related complications were noted.

Clip detachment and coronary embolization are rare but life-threatening complication after TEER.^1^ In this patient, device embolism may due to insufficient leaflet capture at indentation area. Sufficient leaflet capture, early diagnosis, and intervention are key for prevention and treatment for this complication. Percutaneous retrieval of embolized ‘Pascal-like’ clip using two gooseneck snares technique is feasible.

## Supplementary Material

ytae302_Supplementary_Data

## Data Availability

The data underlying this article are available in the article and will be shared with the corresponding author at reasonable request.
